# Ultrasonic Power and Data Transfer through Multiple Curved Layers Applied to Pipe Instrumentation

**DOI:** 10.3390/s19194074

**Published:** 2019-09-20

**Authors:** Victor L. Takahashi, Alan C. Kubrusly, Arthur M. B. Braga, Sully M. M. Quintero, Sávio W. O. Figueiredo, Ana B. Domingues

**Affiliations:** 1 Department of Mechanical Engineering, Pontifical Catholic University of Rio de Janeiro, Rio de Janeiro 22451-900, Brazil; abraga@puc-rio.br (A.M.B.B.); sully@puc-rio.br (S.M.M.Q.); savio@puc-rio.br (S.W.O.F.); 2 Center for Telecommunication Studies, Pontifical Catholic University of Rio de Janeiro, Rio de Janeiro 22451-900, Brazil; alan@cpti.cetuc.puc-rio.br; 3 Shell Technology Department, Shell Brasil Ltd.a, Rio de Janeiro 20031-170, Brazil; A.Domingues@shell.com

**Keywords:** power and data transmission, ultrasonic communication, multiple layers acoustic transmission, passive sensor communication

## Abstract

Ultrasonic power and data transfer through multilayered curved walls was investigated using numerical and experimental analysis. The acoustic channel used in this paper was formed by two concentric pipes filled with water, aiming for applications that involve powering and monitoring sensors installed behind the pipe walls. The analysis was carried out in the frequency and time domains using numerical and experimental models. Power and data were effectively simultaneously transferred through the channel. A remote temperature and pressure sensor was powered and interrogated throughout all the layers, and the power insertion loss was 10.72 dB with a data transmission rate of 1200 bps using an amplitude modulated scheme with Manchester coding. The efficiency of the channel was evaluated through an experimental analysis of the bit error rate (BER) with different values of signal-to-noise ratio (SNR), showing a decrease in the number of errors compared with detection without Manchester coding.

## 1. Introduction

Ultrasonic waves can be used for wireless data communication and power transfer. Typical examples of well-developed practices include underwater acoustic communications [1,2,3], and biomedical applications where ultrasound is used to power and communicate with implantable devices [4,5,6]. The field of power and data transfer through metal layers using ultrasonic waves is, however, less well developed when compared to the aforementioned fields. Wireless transmission of power and data through metallic layers is relevant in applications where metallic walls cannot be penetrated, such as submarines [7] and pressure vessels [8]. Over the past 20 years, the use of an acoustic–electric channel on a metal barrier has been investigated [9], revealing a higher efficiency of power and data transfer when compared to electromagnetic-based solutions, either based on inductive [10,11,12] or capacitive [13] couplings. Several setups that rely on different types of transducers [14], channel configurations [8], and data modulations [10,15,16,17] have been investigated.

The most common setup consists of a pair of ceramic piezoelectric transducers encompassing a metal layer through which communication is intended. Adhesive epoxy is usually used to bond the transducers on the metal surfaces. The power and data communication through an acoustic channel typically contains three major components, namely the outside block, the inside block and the acoustic channel. One possible topology is the so-called reflected-power [18]. In this setting, information is sent from the inside block to the outside block, whilst energy is sent from the outside to the inside block. Communication is possible by varying the impedance of the piezoelectric transducer located at the inside block, which consequently changes the amplitude of the reflected wave [15,17,18], and modulates the continuous wave signal sent from the outside block. Typical values of data rate and power transfer for this configuration are around hundreds of bps and hundreds of mW [9,15,18].

Usually the medium through which ultrasonic communication takes place consists only of a single metallic layer and bonding layers necessary to fix the transducers [9]. It is naturally expected that, if the medium is composed of several layers, then the communication efficiency would be reduced due to the impedance difference along the wave propagating path, which naturally introduces mismatches, and consequently, less power may be expected to cross the channel. There are, however, few published works on ultrasonic communication and power transfer across multilayered channels. Chakraborty et al. [19] analyzed the transmission of power and data in the case of multiple layers of different materials, in a steel–water–steel configuration. Two channels were characterized, the first with a steel thickness of 15.97 mm and a water layer of 88.3 mm, and the second with a steel thickness of 10.92 mm and a water layer of 55.62 mm. They experimentally showed a power transfer efficiency of 30% and a practical achievable data rate around 4 Mbps using orthogonal frequency-division multiplexing (OFDM) modulation.

Another characteristic that directly impacts the efficiency of the ultrasonic wave transmission is the eventual curvature of a layer; a circular pipe or a metal curved shell could change the power and data transfer [17,20]. Analysis and characterization of ultrasonic channels that have curved metal walls is gaining attention. Chase [17] demonstrated the feasibility to transmit power and data through a curved metal wall made of aluminum. A pipe with an outer diameter of 76 mm and thickness of 3.2 mm was experimentally evaluated by attaching piezoelectric transducers on the pipe surface with a transition piece that matched the curvature of the tube.

When the electric impedance of the system was matched, a power transfer efficiency close to 30% and a data transfer rate of 10 kbps were achieved. Ding Xin et al. [20] adopted a similar setup, but used a wider and thicker steel pipe with a 85 mm outer diameter and 10 mm thickness for power transfer only. The latter achieved 28 dB of insertion loss; considerably lower than Chase’s [17] work, which reached 5.7 dB.

A multilayered curved channel can be of interest in applications that concern transmission through concentric pipes. An example of such a scenario is found in the oil and gas industry, and, more specifically, in wellbore monitoring. This case consists of an inner and an outer pipe with liquid between them. The former is known as production tubing and the latter as casing pipe. One is usually interested in monitoring the structural health of regions beyond the casing layers. This can be achieved by placing sensors on the exterior of the casing pipe. Nevertheless, access is only possible in the interior of the tubing. Therefore, the issue is how to power supply the sensors and to get their data. A possible solution is to transfer power and to interrogate the sensor utilizing ultrasonic waves generated in the tubing. This scenario is, however, composed of a curved multilayer structure, being thus more complex than the aforementioned usual applications of ultrasonic through-wall communication. This paper investigates the potential of using a multi-layered structure composed of a water layer and a curved steel barrier aiming for a future wellbore monitoring system. Our experimental setup consisted of an arrangement of a pair of piezoelectric ultrasonic transducers enclosing multiple layers formed by two concentric pipes separated by water. In the inner pipe, a cover made of steel protects the piezoelectric transducer, adding, however, one more acoustic layer to the arrangement. The piezoelectric transducers have a nominal resonance frequency of 1 MHz, and the communication between then uses the impedance variation in order to amplitude modulate the carrier signal [17]. A complementary investigation was carried out by simulating the electro-acoustic channel using a one-dimensional multilayer electro-mechanical model designed in PSpice^®^ [18].

The remainder of the paper is organized as follows: Section 2 reviews power and data acoustic channel (PDAC) systems and the power and used data communication method. Section 3 describes the numerical and experimental acoustic channel analysis. Section 4 describes the system implementation and shows the experimental and numerical results. Section 5 concludes the paper.

## 2. Power and Data Acoustic Channel System Overview

**Figure 1:**
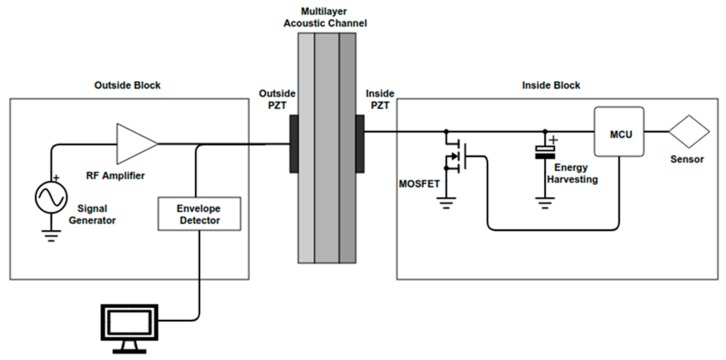
Power and data acoustic channel (PDAC) overall system diagram.

The acoustic channel is composed of three major components, namely the outside block, inside block and acoustic channel, as Figure 1 illustrates. The outside block is composed of a signal generator and a radio frequency (RF) amplifier, both are responsible for sending a carrier signal to an ultrasonic transducer (made of lead zirconate titanate (PZT) ceramic), named the outside transducer, and a circuit to demodulate the received signal, based on an envelope detector. The inside block is composed of a circuit that rectifies and stores the received energy, a piezoelectric transducer (named the inside transducer), and a circuit to modulate the signal. The acoustic channel block is the medium connecting both sides by means of an ultrasonic wave propagating across it. Unlike the conventional application [14,18], in this paper the propagating medium is not composed of a single metallic flat layer. Instead, it presents a multilayer curved structure formed by two concentric tubes separated by water.

### Power and Data Communication Method

The sensors and the complementary electronics that are located on the inside block are completely powered from the outside block through ultrasonic waves that reach the inside block. These acoustic waves are generated in the transmitting transducer, outside the PZT (shown in Figure 1), by a continuous wave (CW) voltage that is applied at the outside the PZT’s terminals (the signal generation and RF amplifier in Figure 1). The waves then propagate and impinge the face of the receiving transducer, inside the PZT (shown in Figure 1), in the form of vibration. Some energy is attenuated in the acoustic channel because of material losses [18,21] and reflection due to an acoustic impedance mismatch between the layers across the channel; however, sufficient power reaches the inside block to supply the electronic components. The transducer in the inside block then converts the vibration to voltage. The electric energy is stored in the inside block in order to power all the necessary electronics and sensors.

The data communication principle is based on the impedance variation [15,16,17,18]. The digital information from the sensors is read by a microcontroller unit (MCU), which encodes it in two logic levels, and sends it to one of its pins. This pin is connected to the metal oxide semiconductor field effect transistor (MOSFET) transistor that short-circuits the terminals of the inside piezoelectric transducer to ground, as shown in Figure 1. By switching the transducer terminals between open and shorted, the acoustic impedance of the channel changes because the electrical terminal of the PZT has its boundary condition altered. Consequently, the equivalent electric impedance seen in the terminals of the outside PZT also, slightly, changes, which consequently varies the CW signal amplitude in the outside PZT’s terminal. Therefore, an amplitude modulation scheme is achieved, and it can be demodulated by the outside block, using a simple envelope detector followed by a digital signal processor in order to interpret the sensors’ data. Therefore, power is sent from the outside block to the inside block and data is sent from the inside block to the outside block using a single channel and carrier wave.

## 3. Acoustic Channel Analysis

### 3.1. Numerical Model

**Figure 2:**
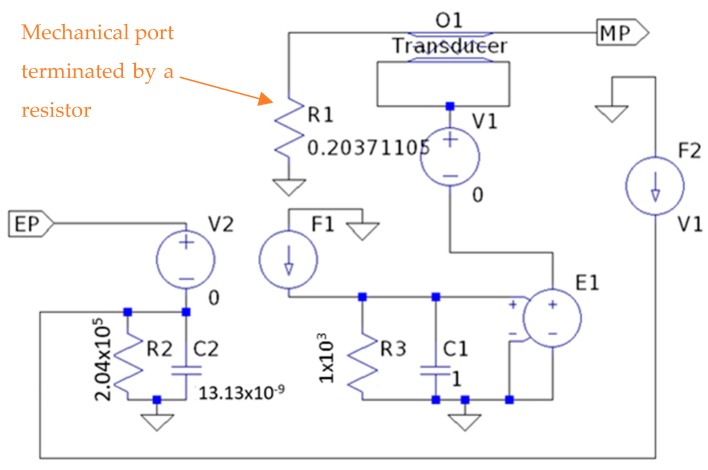
Equivalent electrical model of a piezoelectric ceramic transducer.

The electro-mechanical model used in this paper follows the approach developed by Roa-Prada et al. [20], which was implemented on an electrical circuit simulator. The whole acoustic channel, composed of piezoelectric transducers and intermediate layers, can be simulated by considering the electro-mechanical analogy between voltage and current to pressure and velocity, respectively. The piezoelectric transducer component is modeled as a three-port device, being one electrical and two mechanical (shown in Figure 2). The electric signal is applied or sensed in the electrical port (EP), whereas the two mechanical ports (MP), represent the front and back faces of the transducer, which vibrate when ultrasonic waves are generated or impinge upon them. One of the mechanical ports is terminated by a resistor, simulating the back face of the transducer in air. The intermediate layers are implemented as a purely two-port mechanical device.

**Figure 3:**
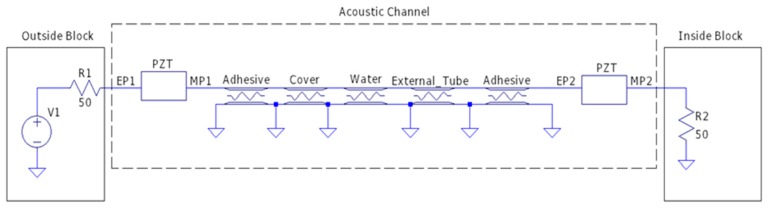
Ultrasonic channel formed by the intermediate layers and piezoelectric transducers. MP denotes mechanical port, whereas EP denotes electrical port. The element labeled PZT is the equivalent model of a piezoelectric transducer.

Figure 3 shows the diagram of the model designed in LTspice^®^. As one can notice, in the acoustic channel block, the intermediate layers are modeled as transmission lines. The power analysis is performed by means of S-parameters, which are typical network parameters that relate the amount of power reflected or transmitted in each pair of ports [22]. In the present case, the S21 parameter of the whole channel indicates how much power a load in the inside block absorbs when a certain amount of power is injected in the outside block. Moreover, this circuit model approach can provide transient analysis such as instant voltage, current and power, which are useful to analyze the data communication system. Therefore, this model helps with understanding some behaviors of the electro-acoustic channel, such as power transfer and data communication, by evaluating the electrical signals on the electrical ports named EP2 and EP1. The material properties can be easily modified by changing the model parameters. An important advantage of the model is that multiple layers can be added or removed by simply connecting/disconnecting the new transmission lines.

### 3.2. Experimental Setup

**Figure 4:**
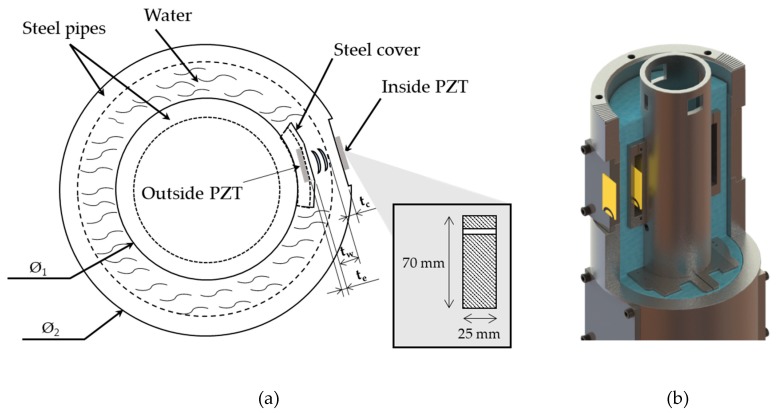
(**a**) Schematic diagram of the experiment: the outside transducer is bonded to the case, and the inside transducer is directly attached to the external pipe; (**b**) 3D isometric view of the experimental setup.

An experimental setup was created in order to investigate power and data transfer across multiple curved layers (water and steel). The setup has two concentric pipes with water in between them with dimensions shown in Figure 4a,b, respectively. The geometric parameters such as diameters and thickness of the pipes are based on the actual dimensions used in wellbore systems. For a proper operation, the piezoelectric transducers must be aligned to reduce the insertion loss of the acoustic channel [17,18]; this means that the energy transfer to the inside block is dramatically dependent on the alignment of both transducers. According to this, and knowing that the control of alignment between the transducers is difficult for a wellbore application, a higher area of the piezoelectric transducer was used to reduce the loss of power due to misalignment. The dimensions of the rectangular-shaped piezoelectric transducers were 70 mm × 25 mm × 2 mm, and are also shown in the inset plot of Figure 4a. Furthermore, generally the acoustic channel has a high selectivity in frequency [16,17,18], meaning that the system must operate in an optimum frequency that provides sufficient power to the inside block and an adequate voltage variation to allow a reliable communication. As it can be seen in Figure 4b, the experimental setup comprised a flat stainless steel case that covers the internal transducer, keeping its electrodes from being in contact with the water and also leaving the piezoelectric back face in contact with air. This case is 7.5 mm thick and is attached on the internal pipe. The water layer and the external pipe section are 20.5 mm and 20 mm thick, respectively. The external and internal pipes are made of stainless steel. The physical properties of the different materials used in the experimental setup are shown in Table 1. It is worth noticing that, despite the whole structure being cylindrical in nature, the PZTs were bonded to flat surfaces in order to provide better coupling for generated and received vibrations, as shown in Figure 4. Consequently, in what concerns the ultrasonic propagating path, there is only one curved interface in the channel, namely the interface at the inner radius of the outer steel pipe and the water annulus, as can be seen by the outer dashed circle in Figure 4a.

### 3.3. Power and Data Communication Analysis

In order to evaluate the power and data communication of the experimental setup, a vector network analyzer (VNA) was used. The S21 scattering parameter was measured and used to evaluate the power transfer. With this equipment, it is also possible to measure the input impedance of the system. Therefore, from the curves of impedance for the two cases, open and shorted, the input voltage variation (*VV*) was calculated using Equation (1), through the difference of the input voltage when varying the input impedance

(1) $$VV=\frac{\left(\left|Z_{o}-Z_{c}\right|\right)}{\left(50+Z_{o}+Z_{c}\right)+\left(Z_{o}\times Z_{c}\right)/50}\times V,$$

where *Z_o_* is the input impedance when the transducer is open-circuited, *Z_c_* is the input impedance when the transducer is short-circuited, and *V* is the voltage applied to the transducer. Equation (1) is obtained by considering that the input voltage in each case is provided by a voltage divider circuit between a 50 Ω nominal impedance and the input impedance. One can notice that the amplitude of the input voltage variation is proportional to the applied voltage *V* and the impedance difference $\left(\left|Z_{o}-Z_{c}\right|\right)$. A 50 Ω resistor, the output impedance of the VNA, is used in the calculation.

**Figure 5:**
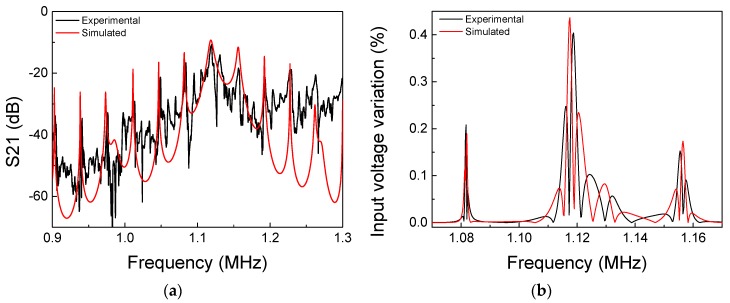
(**a**) Power transfer analysis and (**b**) input voltage variation of experimental and simulated results.

Figure 5a,b show the experimental and numerical results for the S21 scattering parameter and the input voltage variation, respectively. As it can be seen, there are several peaks in Figure 5a due to the wave reverberation within the channel caused by reflections in between the layers and at the ends of the channel, thus creating several resonance frequencies in the working bandwidth, as commonly occurs in this type of ultrasonic communication system [9,21]. The simulated S21 curve of Figure 5a reasonably matches the more pronounced peaks of the experimental curves. Regarding the power transfer, the main maximums occur at (1.118 MHz; −9.28 dB) and (1.119 MHz; −10.79 dB) for the simulated and experimental curves, respectively, resulting in a difference of 1 kHz in the frequency and 1.51 dB of the insertion loss. This amplitude difference may be due to differences between the actual and the tabled material properties used in the numerical model. Additionally, it is also possible to observe that the experimental curve has additional peaks with lower amplitudes between the multiple resonances of the channel that might be caused by other ultrasonic modes of propagation created inside the channel. It is also worth noticing that the simulated model, however, does not include the characteristics of a curved wall because it was developed to contemplate only one-dimension wave propagation [23,24]. With respect to the input voltage variation shown in Figure 5b, the main maxima occur at (1.118 MHz; 0.43%) and (1.119 MHz; 0.40%) for the simulated and experimental curves, respectively. In this case the difference between the input voltage variations is 0.03%. It is straightforward to conclude that a good operating point is at 1.119 MHz as it represents an operating point where both power transfer and data communication are augmented. It is worth noticing that if the propagating distance were larger, lower S21 values would be expected due to the higher attenuation along the channel. Low S21 is, obviously, an issue for power transfer because lower power arrives at the inside block. However, it is also complicating for data transmission even though communication relies on the difference of input voltage. The latter holds due to noise; if the absolute level is too low, the voltage variation may be undetectable. Thus, the propagation distance may be a limiting factor of this method if very long.

## 4. System Implementation

The electro-mechanical model presented in Section 3 is used to evaluate the wireless power transfer and data communication. An equivalent circuit simulates the electronic devices of the inside and outside blocks, and the multilayer acoustic channel, as illustrated in Figure 3. The outside block contemplates a signal source connected to an amplifier, and a demodulation circuit based on an envelope detector. The inside block, on the other hand, has a voltage doubler, a voltage regulator to supply the electronic components, and a MOSFET to perform the data communication.

### 4.1. Full System Simulation

**Figure 6:**
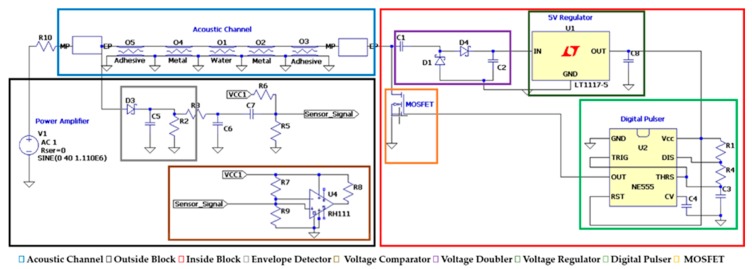
Overall LTSpice schematic system.

**Figure 7:**
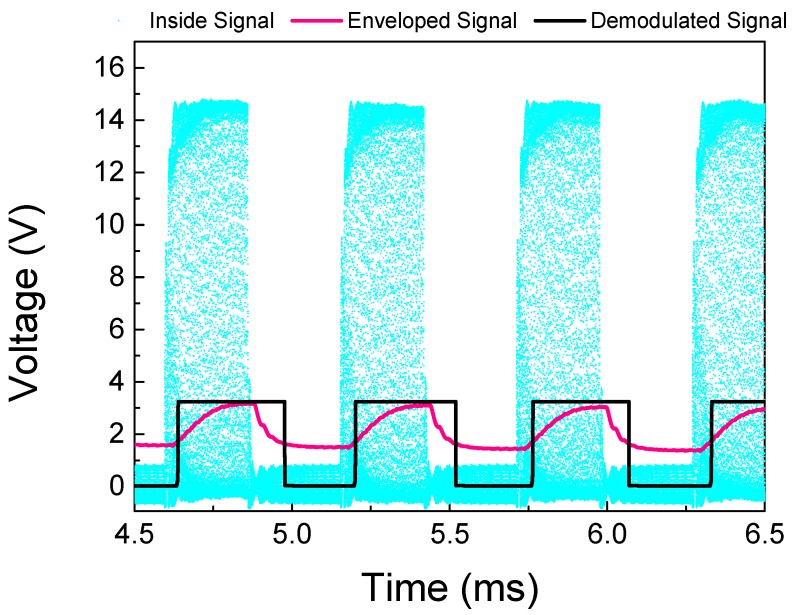
Results of the electrical model.

The full system implementation designed on LTSpice, Figure 6, was implemented based on the experimental setup described in Section 3. The electrical signal applied to electrodes of the outside piezoelectric transducer was set to operate with an amplitude of 40 Vpp at 1.119 MHz, where the maximum power transfer and voltage variation occur, according to Section 3.1. The whole system simulation is shown in Figure 6. In the outside block, in black, an envelope detector and a voltage comparator, in gray and brown, respectively, were designed. The output signal represents the data transmitted from the inside block. The isolated part of the system, the inside block, ensembles the components to conditionate the received power, the voltage doubler circuit, and the voltage regulator, in purple and dark green, respectively. The other components simulate the data transmission, here chosen as a digital pulser and n-channel MOSFET, in light green and orange, respectively. The digital pulser has a transmission rate of 4000 bps and the output is directly connected to the MOSFET gate. Figure 7 shows three waveforms acquired from a full-system simulation, where the cyan one represents the voltage that is received by the inside transducer, the purple is the signal after the carrier signal passes through the envelope detector, and lastly, in black, the demodulated signal, which is the digital data when the enveloped signal passes through the voltage comparator. The generated voltage on the inside transducer has a maximum baseline of approximately 14 V, and reaches around 1 V when the MOSFET is conducting. Due to the use of non-ideal elements implemented in the simulation, the minimum baseline voltage has a non-null voltage, as expected from the MOSFET on-resistance. The enveloped signal varies by approximately 1.5 V after passing through the envelope detector circuit, while the demodulated signal has a squared waveform of the same frequency and amplitude of the digital pulser.

### 4.2. Multilayer Curved Wall Full System Experiment

**Figure 8:**
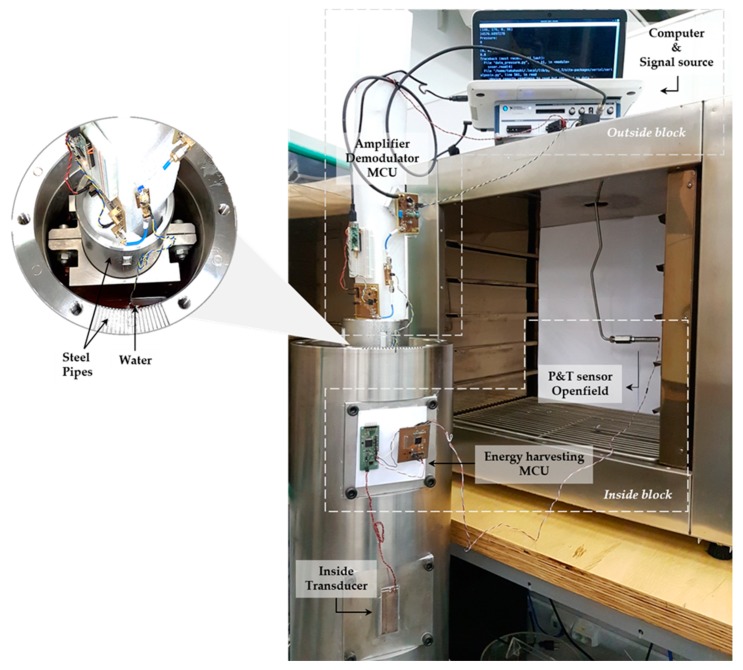
Experimental setup.

Figure 8 shows the experimental setup. The inside and outside blocks, as well as the other components, are highlighted. In the inside block, a pressure and temperature sensor from Openfield replaces the digital pulser of the diagram in Figure 6 and a low-power microcontroller, MSP430F2619 from Texas Instruments^®^, Thief River Falls, MN, USA, acquires the data of the sensor and generates asynchronous information based on Manchester coding [25]. The sensor, located in the inside block, is interrogated once per second, characterizing a low-power mode. The energy storage circuit guarantees the necessary power for the inside block when the transistor is in the on-state. The outside block has an envelope detector circuit and a digital signal processing code implemented inside the MCU to conclude the demodulation of the sensor data. The carrier frequency of the system is generated using a signal generator NI-VirtualBench model 8012, and amplified by a power amplifier before driving the outside piezoelectric transducer. After rebuilding the digital information of the sensor, the data is sent to a computer via a serial port.

**Figure 9:**
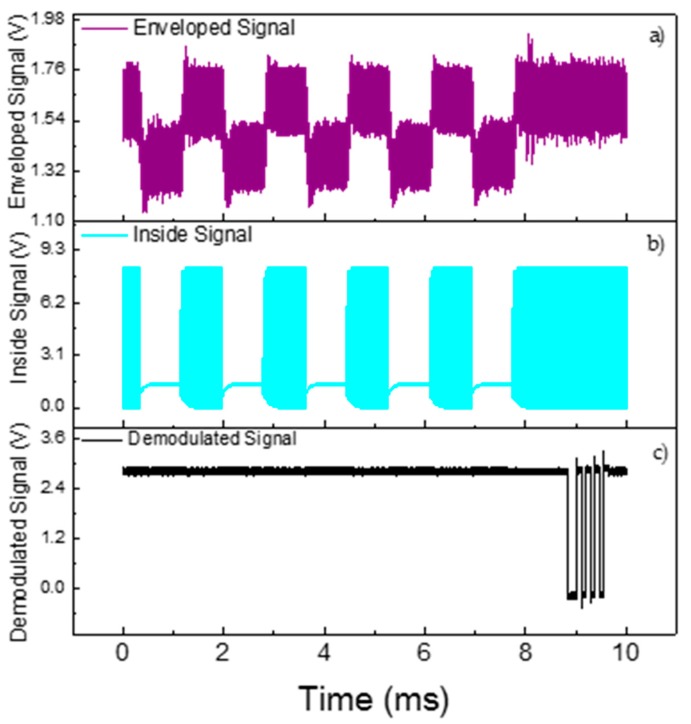
Oscilloscope signals showing (**a**) the enveloped signal after the envelope circuit detector in purple (500 mV/div), (**b**) the inside voltage signal that reaches the inside transducers in cyan (5 V/div) and (**c**) the demodulated signal processed inside a MCU and transmitted to an output pin (5 V/div).

The voltage signals shown in Figure 9 were measured using a digital oscilloscope model MDO3024 from Tektronix^®^. A typical voltage signal produced on the inside block by the inside transducer is presented in Figure 9b. Such as what occurred in the simulation analysis, the voltage has two levels depending on the state of conduction of the MOSFET. These two levels correspond to the sensor data rate of 1200 bit/s. The high level baseline is approximately 10 V; sufficient to power the electronic components even when the energy supply is almost null due to the MOSFET short-circuited state. The outside signal in purple in Figure 9b is the voltage after the envelope detector circuit. This is the first stage of demodulation, and typical values of the difference in voltage amplitude range from 150 mV to 500 mV, approximately, depending on the input voltage.

The transmission of the sensor data between the inside and the outside is implemented using amplitude shift keying (ASK) modulation [25], varying the impedance of the internal transducer. The data flows in only one direction, from the inside to the outside. However, the reverse direction would also be possible, working as a half-duplex, by varying the amplitude of the carrier in such a way to identify which side is transmuting, either inside or outside.

In the reception, the demodulation is implemented using a non-coherent circuit, which means that the modulated signal passes through an envelope detector and a low-pass filter. In most common circuit demodulations, a comparator with a threshold level reconstructs the digital signal; however, in our system, the amplitude variation of the demodulated signal is often low, approximately 300 mV. Other characteristics of the system that affect detection capability, other than the low signal, include the dynamic DC level of the signal in the reception. In the idle mode, when no bits are being transmitted, the DC level is constant; however, when the inside block starts streaming the data, the direct current (DC) level decreases to balance the signal and therefore would require different threshold levels for a proper demodulation. The system is also affected by the amount of power that reaches the inside block, which is directly related to the power capacity to activate the MOSFET. These characteristics make it difficult to have a static threshold level on the comparator, increasing the error detection. The solution, therefore, was to use Manchester coding, which is less susceptible to noise and variations in DC level data. Thus, after passing through the envelope detector and low pass filter, the signal is inserted into the analog–digital converter (ADC) microcontroller to complete the second stage of demodulation using a digital processing unit. A sampling rate of 60 kS/s and a moving average of four samples of unwanted noise present after the envelope detector is effectively removed was used, making it possible to demodulate signals with poor signal-to-noise ratio. Furthermore, the use of Manchester coding favors the digital demodulation inside the MCU by using edge transition instead of level voltage to demodulate each bit. The black signal in Figure 9c is the reconstructed sensor data measured at the on-output pin of the microcontroller after the enveloped signal has been processed. The digital signal has a speed of 115 kbps in the universal asynchrounous receiver/transmitter (UART) protocol, and it can be connected to any computer or data analyzer using UART-USB converters.

### 4.3. SNR and BER Analysis

In this section an experimental procedure was performed to analyze the data transmission error as a function of the signal-to-noise ratio (SNR). The quantity of error in the reception was acquired by comparing the raw data before the transmission through the acoustic channel, and the received signal. Both raw and received data were bit compared one-by-one, which gives the bit error rate (BER), where the data rate was fixed at 64 bps. The SNR was measured by separating the energy of the signal and the energy of the noise. The signal, measured after the envelope detector circuit, contains the sum of the data signal and noise; however, as the raw data acquired on the transmitter mostly has the energy of the data signal, the noise energy is isolated through a fast Fourier transform (FFT) operation, removing the raw data frequency bins of the data signal. The missed frequency bins, from the noise signal, are therefore interpolated to have all of the frequency components. The amplitude levels of the signal data are summed, where the DC component is removed, and divided by the sum of the noise amplitudes, approximately estimating the SNR.

For a fixed SNR, the quantity of the bit error was acquired. The SNR was varied over different values of frequency instead of varying the input voltage of the power amplifier, and so maintaining almost the same noise level. One can notice that slightly changing the operating frequency does not interrupt the functionality of the system, but changes the input voltage variation as shown in Figure 5b. The frequency variation, therefore, increases or decreases the energy of the signal data in the reception.

**Figure 10:**
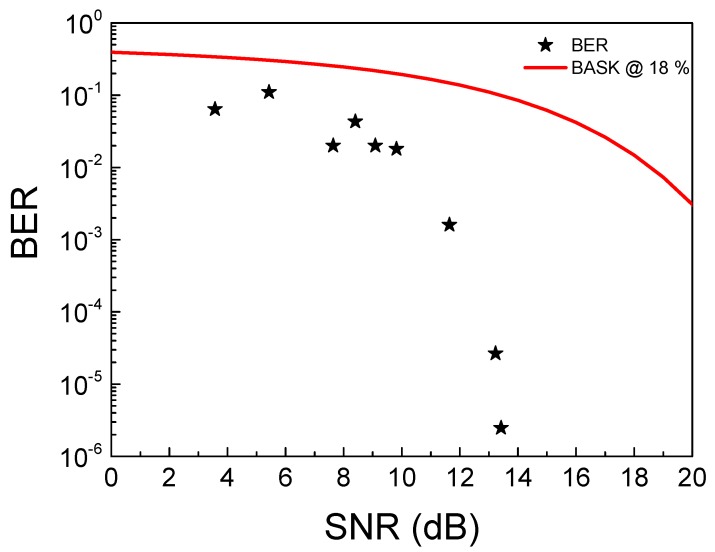
Experimental and theoretical BER curves.

**Figure 11:**
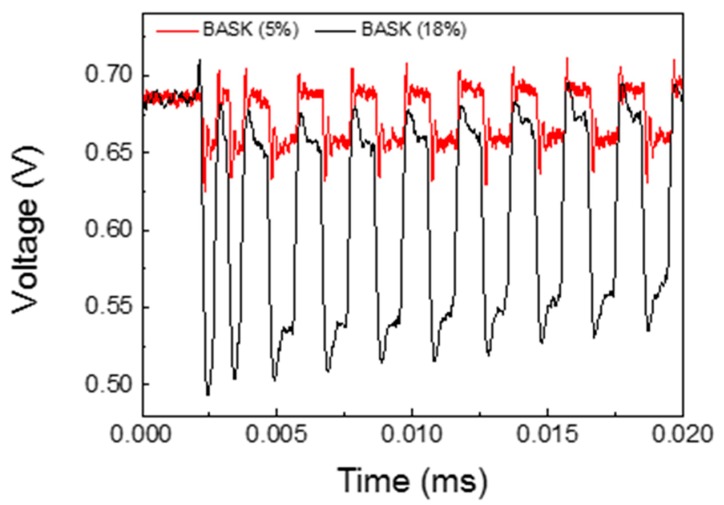
Received signal for BASK 5% and BASK 18%.

Figure 10 shows the experimental and theoretical results of BER versus SNR. The theoretical curve, resulting from Equation (2), represents the probability of a bit error rate for a BASK modulation when the variation between the high level and the low level, bit 1 and 0, respectively, is 18% [26]. This variation represents the best value in our experimental results, approximately at 13.5 dB with 10^−6^ of bit error rate. The experimental analysis also shows the results when the voltage variation between the high and low level is 5%, at approximately 3 dB with 10^−1^ of bit error rate. Both signals are shown in Figure 11. In any case, it should be noted that, for our system, the error data rate is lower than the theoretical curve due to Manchester coding implementation.

(2) $$P_{e}\left(\mathrm{BASK}\;\left(18\%\right)\right)=\frac{1}{2}erfc\left(\sqrt{\frac{3\mathrm{SNR}}{80}}\right)$$

## 5. Conclusions

Ultrasonic power and data were successfully transmitted through a multi-layered curved structure that was designed for wellbore monitoring. The pipe’s geometry was based on the actual dimensions used in the field, as well as the direction of power and data. Power was sent from the inner pipe to the outer pipe, whereas data went on the reverse path, which is needed for wellbore monitoring systems. The experimental results showed the capability of the system to transfer the necessary energy to supply the electronics on the inside block, which, at full operating capability, has a power consumption of approximately 100 mW, and also the capability to communicate with a digital sensor at a rate of 1200 bps using Manchester coding. The proposed solution also suggested that any digital sensor can be wirelessly interrogated through the acoustic channel. The implementation of Manchester coding and the MCU also allowed the system to operate in a noisy condition compared with the detection method of the analog voltage system. The system was able to demodulate the sensor information with extremely low values of the enveloped signal (approximately 150 mV) when using a digital low pass filter implemented on the MCU. The proposed method is, therefore, promising for field applications with wellbore monitoring systems.

In order to increase the comprehension of the acoustic channel behavior, future studies on how the misalignment between transducers and temperature affect the power and data transmission of the system need to be conducted. Furthermore, the use of techniques to increase the efficiency, such as electrical impedance matching and other types of modulation to increase the channel capability, need to be investigated.

## Figures and Tables

**Table 1:** Material properties.

Piezoceramic: PZT5			Fluid: Water (20 °C)		
Density	ρ (kg/m³)	7733	Density	ρ (kg/m³)	1000
Sound speed	υ (m/s)	4419	Sound speed	υ (m/s)	1484
Quality factor	Q_m_	48	Acoustic damping coefficient	α_v_ (Np/m)	0
Electrical permittivity	ε_33_^s^_/_ε_0_	1101	Thickness	t_w_ (m)	20.5 × 10^−3^
Piezoelectric coupling factor	κ_33_	0.58	Area	A (m²)	175 × 10^−3^
Piezoelectric coefficient	C_33_ (N/m²)	15.1 × 10^10^			
Area	A (m²)	175 × 10^−3^			
Thickness	t (m)	2 × 10^−3^			
**Metal: Stainless Steel**			**Adhesive: Epoxy**		
Density	ρ (kg/m³)	7894	Density	ρ (kg/m³)	1400
Sound speed	υ (m/s)	5619	Sound speed	υ (m/s)	2344
Acoustic damping coefficient	α_v_ (Np/m)	4	Acoustic damping coefficient	α_v_ (Np/m)	172
Area	A (m²)	175 × 10^−3^	Area	A (m²)	175 × 10^−3^
Outer pipe thickness	t_O_ (m)	7.5 × 10^−3^	Thickness	t (m)	150 × 10^−6^
Steel cover thickness	t_e_ (m)	20 × 10^−3^			
